# A Therapeutic Antibody against West Nile Virus Neutralizes Infection by Blocking Fusion within Endosomes

**DOI:** 10.1371/journal.ppat.1000453

**Published:** 2009-05-29

**Authors:** Bruce S. Thompson, Bastiaan Moesker, Jolanda M. Smit, Jan Wilschut, Michael S. Diamond, Daved H. Fremont

**Affiliations:** 1 Pathology and Immunology, Washington University School of Medicine, St. Louis, Missouri, United States of America; 2 Department of Medical Microbiology-Molecular Virology, University Medical Center Groningen, University of Groningen, Groningen, The Netherlands; 3 Medicine, Washington University School of Medicine, St. Louis, Missouri, United States of America; 4 Molecular Microbiology, Washington University School of Medicine, St. Louis, Missouri, United States of America; Institut Pasteur, France

## Abstract

Defining the precise cellular mechanisms of neutralization by potently inhibitory antibodies is important for understanding how the immune system successfully limits viral infections. We recently described a potently inhibitory monoclonal antibody (MAb E16) against the envelope (E) protein of West Nile virus (WNV) that neutralizes infection even after virus has spread to the central nervous system. Herein, we define its mechanism of inhibition. E16 blocks infection primarily at a post-attachment step as antibody-opsonized WNV enters permissive cells but cannot escape from endocytic compartments. These cellular experiments suggest that E16 blocks the acid-catalyzed fusion step that is required for nucleocapsid entry into the cytoplasm. Indeed, E16 directly inhibits fusion of WNV with liposomes. Additionally, low-pH exposure of E16–WNV complexes in the absence of target membranes did not fully inactivate infectious virus, further suggesting that E16 prevents a structural transition required for fusion. Thus, a strongly neutralizing anti–WNV MAb with therapeutic potential is potently inhibitory because it blocks viral fusion and thereby promotes clearance by delivering virus to the lysosome for destruction.

## Author Summary

Antibodies are essential components of the immune response against many pathogens, including viruses. A greater understanding of the mechanisms by which the most strongly inhibitory antibodies act may influence the design and production of novel vaccines or antibody-based therapies. Our group recently generated a highly inhibitory monoclonal antibody (E16) against the envelope protein of West Nile virus, which can abort infection in animals even after the virus has spread to the brain. In this paper, we define its mechanism of action. We show that E16 blocks infection by preventing West Nile virus from transiting from endosomes, an obligate step in the entry pathway of the viral lifecycle. Thus, a strongly inhibitory anti–West Nile virus antibody is highly neutralizing because it blocks fusion and delivers virus to the lysosome for destruction.

## Introduction

Neutralizing antibodies can inhibit virus infection by impeding one of several critical steps of the virus lifecycle. These include blocking attachment to the cell surface, interaction with host factors required for internalization, and structural transitions on the virion that drive membrane fusion (reviewed in [1],[2]). Antibodies can independently neutralize virus infection by promoting virus aggregation, destabilizing virion structure, and blocking budding or release from the cell surface (reviewed in [3]). Historically, many of the most potently neutralizing antibodies inhibit infection by interfering with required interactions between viruses and obligate cellular receptors (e.g., rhinovirus and ICAM-1, HIV and CD4 or CCR5, and poliovirus and CD155).

West Nile virus (WNV) is a mosquito-borne positive polarity RNA virus of the Flavivirus genus within the *Flaviviridae* family. Similar to other Flaviviruses, such as Dengue (DENV), yellow fever, and Japanese encephalitis viruses, WNV has an ∼11 kb RNA genome that encodes three structural (C, prM/M and E) and seven non-structural (NS1, NS2a, NS2b, NS3, NS4a, NS4b, and NS5) proteins that are generated by cleavage from a single polyprotein [4],[5]. WNV has spread globally and epidemic outbreaks of encephalitis now occur annually in the United States. Infection with WNV causes syndromes ranging from a mild febrile illness to severe neuroinvasive disease and death [6],[7]. There is currently no approved vaccine or therapy for WNV infection.

Structural analysis of the WNV and DENV virions by cryo-electron microscopy [8],[9] reveals a ∼500 Å mature virion with a smooth outer surface. The 180 copies of the E glycoproteins lay relatively flat along the virus surface as anti-parallel dimers in three distinct symmetry environments. Following exposure to low pH in the endosomal compartment, the E proteins rearrange from homodimers to homotrimers, exposing a fusion peptide, which interacts with the endosomal membrane and allows uncoating and nucleocapsid escape into the cytoplasm [10].

The atomic structure of the surface E glycoprotein has been defined by X-ray crystallography for DENV, WNV, and tick-borne encephalitis virus (TBEV) [11]–[15], revealing three conserved domains. Domain I (DI) is a 10-stranded β-barrel and forms the central structural architecture of the protein. Domain II (DII) consists of two extended loops projecting from DI and contains the putative fusion loop (residues 98–110), which participates in a type II fusion event [10],[16],[17]. In the mature virus, the fusion loop packs between two anti-parallel dimers and is solvent inaccessible, protecting the virus from premature fusion and inactivation. Domain III (DIII) is located on the opposite end of DI, forms a seven-stranded immunoglobulin-like fold, and has been suggested as a receptor binding site [18]–[20].

The humoral immune response controls WNV pathogenesis as mice lacking B cells are highly vulnerable to lethal infection [21]. During infection with flaviviruses, most neutralizing antibodies are directed against the E protein, although a subset binds the prM protein [22],[23]. To better understand the structural basis of antibody protection against WNV, we recently generated a large panel of monoclonal antibodies (MAbs) against WNV E protein [24]. One antibody, E16, was observed to block WNV infection in vitro and in vivo and was effective as a post-exposure therapy even 5 days after infection [24],[25]. Potent E16 neutralization occurs with strikingly low stoichiometric requirements, as a virion occupancy of ∼25% is sufficient to inhibit infection [26]. Herein, we determine the mechanism by which this therapeutic MAb neutralizes WNV infection. E16 traffics with WNV particles into permissive target cells, and is strongly inhibitory because it blocks pH-dependent fusion, a critical step in the entry pathway of this virus.

## Results

### MAb E16 does not block WNV entry

**Figure 1:**
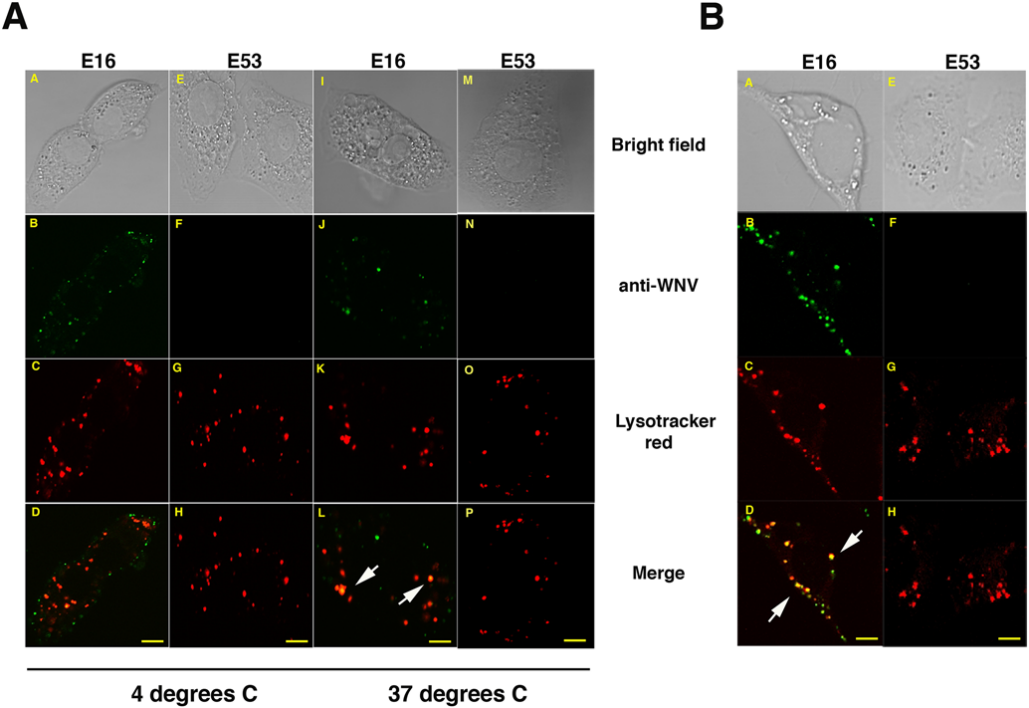
E16-opsonized WNV enters Vero cells. (A) Vero cells were incubated with WNV at an MOI of 100 in the presence or absence of 100 μg/ml Alexa 488-E16 or E53 MAbs. Lysotracker red (50 nM) was added to the cells for the last 30 min of the incubation prior to paraformaldehyde fixation. Green staining indicates Alexa 488 conjugated anti-WNV MAbs (panels B, F, J, and N), red staining indicates lysotracker red dye (panels C, G, K, and O), and yellow staining represents co-localization as reflected by the merged images (panels D, H, L, and P). Cells were incubated for 2 h on ice, washed, fixed, and observed by confocal microscopy (panels A–H). Cells were shifted to 37°C following the incubation on ice, fixed after 15 min and observed by confocal microscopy (panels I–P). White arrows indicate examples of co-localization of anti-WNV MAbs and lysotracker red dye. (B) Cells were infected for 3 h at 37°C in the presence of 100 μg/ml E16 (B, D) or E53 (panels F and H) and lysotracker dye (panels C, D, G, and H) fixed, and analyzed by confocal microscopy. The scale bars represent 10 μm.

A common mechanism of antibody-mediated neutralization of viral infection is to prevent attachment and entry into target cells. Previously published studies suggested that E16 did not dramatically reduce WNV binding to Vero cells but instead inhibited at a post-attachment step [27]. To gain further insight as to how E16 inhibits infection, WNV was pre-incubated with Alexa-488 conjugated E16 or E53, a second inhibitory MAb that binds to the fusion loop in DII, prior to a cell binding assay at 4°C. Subsequently, cells were washed at 4°C, fixed and visualized by confocal microscopy. At 4°C, enveloped viruses, including flaviviruses, remain on the cell surface and are not internalized [28]–[30]. As expected, in the absence of WNV, labeled E16 and E53 were not visualized on the surface or interior of cells (data not shown). When Alexa-488-E53-WNV complexes were added, no fluorescence signal was observed on the surface of Vero cells (Figure 1A, panels F and H), suggesting that E53, as hypothesized previously [27], primarily inhibits WNV attachment to Vero cells. Similar results were obtained with Alexa-488 conjugated E60, a MAb that binds to a similar epitope as E53 in DII (data not shown). In contrast, staining was apparent on the surface of cells incubated with labeled Alexa-488-E16-WNV complexes. Thus, despite saturating and neutralizing concentrations (100 μg/ml) of E16 MAb, WNV binding to Vero cells still occurred (Figure 1A, panels B and D). Analogous results were obtained with the strongly neutralizing DIII-specific E24 MAb (data not shown).

To determine if the E16 MAb restricted virus entry, Vero cells were warmed to 37°C after MAb-WNV complex pre-binding at 4°C, and again visualized by confocal microscopy. As anticipated, Alexa-488-E53-WNV complexes were not detected inside cells (Figure 1A, panels N and P). In contrast, Alexa-488-E16-WNV complexes readily entered cells and accumulated in acidic vesicles that were identified with a pH sensitive dye (Figure 1A, panels J and L). Even after several hours of incubation, E16-WNV complexes remained localized in these acidic cellular compartments (Figure 1B, panels B–D), whereas E53-WNV complexes were not detected within the cells (Figure 1B, panels F–H). In contrast, in the absence of neutralizing antibodies, WNV infection progresses rapidly as demonstrated by the accumulation of E protein in the cell over time (Figure S1).

### E16 blocks infection in a manner analogous to inhibiting endosome acidification

**Figure 2:**
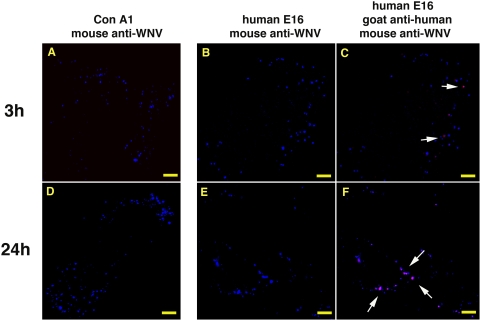
Blockade of endosomal acidification with concanamycin A1 mimics treatment with E16. Vero cells were infected at an MOI of 100 in the presence of 10 nM concanamycin A1 (panels A and D) or 100 μg/ml hE16 (panels B, C, E, and F) for 3 h (panels A–C) or 24 h (panels D–F) and then fixed. Cells were then stained with a pool of Alexa-488 conjugated mouse anti-E MAbs (blue; A-F) and Alexa-647-conjugated goat anti-human IgG (red; C and F) as indicated. Cells were analyzed by confocal microscopy. The white arrows indicate co-localization of hu-E16 and the oligoclonal pool of mouse anti-E MAbs. Representative images are shown from one of two independent experiments. The images were analyzed using the LSM510 confocal microscopy software to assess the overlap in staining. Because of the overlay appearance, we converted the fluorescence images into blue (Alexa-488) and red (Alexa-647) colors for Figure display. The scale bars represent 10 μm.

Because E16-WNV complexes co-localized with an acidified intracellular compartment for several hours, we hypothesized that this MAb prevented virus fusion with endosomal membranes. Because WNV infection requires a pH-dependent structural rearrangement of E proteins for fusion, we evaluated whether concanamycin A1, a vacuolar-ATPase inhibitor [31], blocked WNV infection at a similar cellular stage as did E16. Vero cells were infected at a high multiplicity of infection (MOI) in the presence of 10 nM concanamycin A1 or humanized E16 (hu-E16, 100 μg/ml) or a media control for 3 h or 24 h at 37°C. Cells were washed, fixed, and stained for WNV using an oligoclonal pool of mouse MAbs against the E protein. Samples treated with hu-E16 were also stained with an anti-human IgG secondary antibody to confirm that hu-E16 co-localized with the virus. In the absence of concanamycin A1 or hu-E16, infected Vero cells showed strong staining of E protein at 3 h that was markedly increased at 24 h (Figure S1). Treatment with 10 nM concanamycin A1 resulted in a punctate pattern of E protein staining at 3 and 24 h, suggesting that WNV localized to and likely remained sequestered in endocytic compartments (Figure 2, panels A and D). Analogous to treatment with concanamycin A1, hu-E16-opsonized WNV showed a similar staining pattern up to 24 hours after infection (Figure 2, panels B and E). As co-staining of oligoclonal mouse anti-E protein and hu-E16 was observed over time, it is likely that E16 was still bound to WNV, and these virus-MAb complexes accumulated in endosomal/lysosomal compartments (Figure 2, panels C and F). Of note, in Figure 2C, only a subset of the blue spots (which indicates the presence of the virion) co-stain with hu-E16. This is likely a sensitivity of detection issue as E16 neutralizes infection at both low (∼25% or 30 copies per virion) and high occupancy [26]. Because of the high MOI used, some viruses will be more completely decorated (and thus fluorescent), whereas others will bind fewer antibodies yet still be neutralized. Virions that bind fewer E16 antibodies yet still are neutralized may co-stain less brightly in this microscopic assay.

### E16 blocks fusion at the plasma membrane

**Figure 3:**
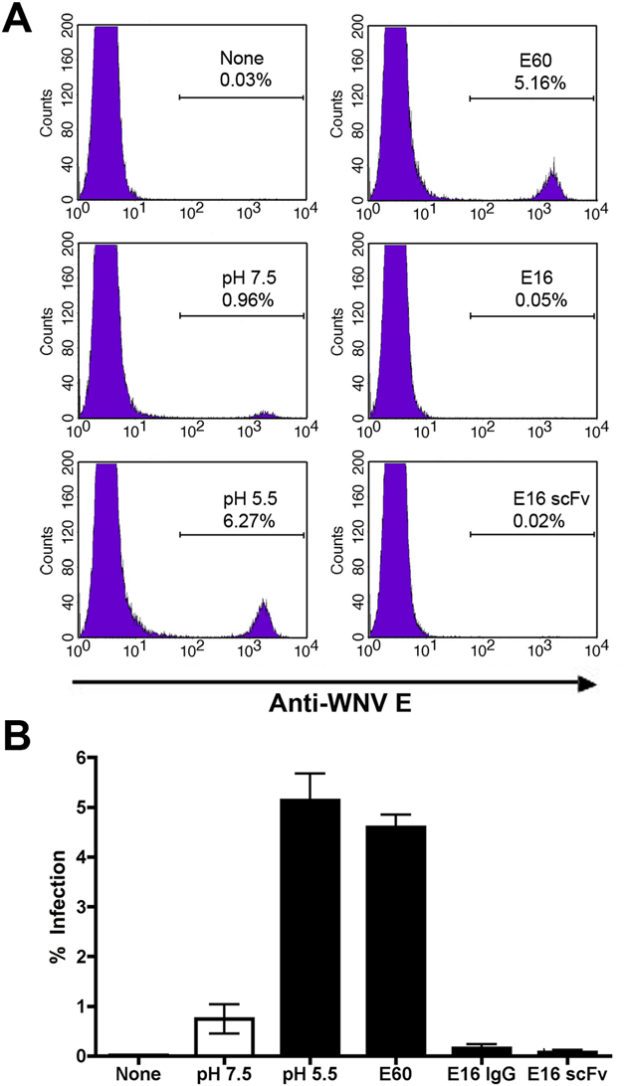
E16 scFv or IgG blocks infection in a plasma membrane fusion assay. WNV (10^6^ PFU) was bound to Vero cells for 2 h on ice after pretreatment with 10 nM concanamycin A1. Subsequently, media, 100 μg/ml E16 IgG, E16 scFv or E60 IgG was added for 30 min on ice, and then the pH shifted at 37°C to pH 7.5 or pH 5.5 for ∼7 min. Cells were washed, the pH normalized, incubated at 37°C for ∼18 h, permeabilized and stained with an oligoclonal pool of anti-E MAbs. The level of infection was assessed by flow cytometry. (A) Representative flow cytometric histogram plots from each condition are shown. The plots are gated to show the percentage of cells that stained positive with an anti-WNV E MAb. The treatment and percentage of positive cells are shown in the top right corner of each plot. (B) The data averaged from three independent experiments is shown with error bars indicating standard deviations. Statistically significant differences between different experimental conditions are described in the text.

The ability of E16 to block WNV egress from endosomes suggested that this MAb directly inhibited the pH-dependent fusion step. Initially, to test this, we used a surrogate plasma membrane fusion infection assay that has been validated for alphaviruses and flaviviruses [32],[33]. Normally, flaviviruses enter cells via receptor-mediated endocytosis, with fusion occurring from within acidic endosomes [29],[34],[35]. However, flaviviruses also can be induced to fuse directly with the plasma membrane, at low efficiency, when cell-bound virus is exposed to an acidic solution [32]. To assess the effects of E16 on virus-plasma membrane fusion, WNV was pre-bound to Vero cells at 4°C, and subsequently incubated on ice with saturating concentrations of E16 IgG, E16 single chain Fv (scFv), E60 IgG, or no MAb. Cells were warmed to 37°C in pH 5.5 media (or pH 7.5 media as a negative control) to induce virus-plasma membrane fusion and analyzed at 24 hours for level of infection by flow cytometry. In all experiments, 10 nM concanamycin A1 was added to inhibit infection via the canonical receptor-mediated endocytic pathway. As expected, in the absence of antibody, addition of media at neutral pH (7.5) did not promote productive infection (∼0.7% WNV antigen^+^ cells, Figure 3A and 3B). Exposure of cell bound WNV to media at pH 5.5 resulted in a ∼7 fold increase in infection (∼5.1% WNV antigen^+^ cells, P<0.0005, Figure 3A and 3B). The addition of E60 following viral attachment did not appreciably affect virus-plasma membrane fusion (P = 0.4), confirming earlier results that this MAb does not inhibit Vero cell infection at a post-attachment step [27]. In contrast, both E16 IgG and scFv efficiently blocked WNV-plasma membrane fusion (0.15% and 0.08% WNV antigen^+^ cells, respectively; Figure 3A and 3B, P<0.0001).

### E16 blocks pH-dependent fusion of WNV with liposomes

**Figure 4:**
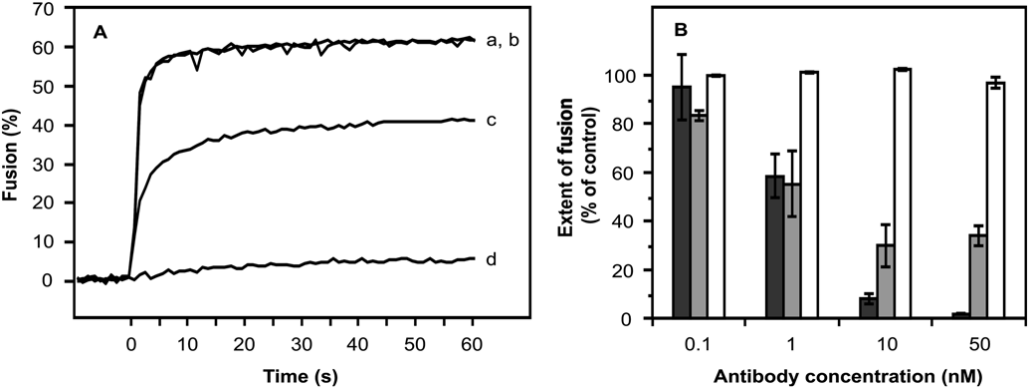
E16 blocks low pH-induced fusion of WNV with liposomes. Fusion of pyrene-labeled WNV with liposomes at pH 5.4. Fusion was measured in real-time as described in the Materials and Methods. (A) (a) no antibody; (b) 0.1 nM E16; (c) 1 nM E16; and (d) 50 nM E16. Representative viral fusion curves are from at least three independent experiments. (B) Effect of different concentrations of MAbs on WNV-liposome fusion. The WNV-liposome fusion profiles are shown as a percentage of the control (pH 5.4, without MAbs). Black bars, E16; gray bars, E60; and white bars, E111. Data are expressed as the mean of at least three independent experiments and the error bars indicate standard deviations.

To confirm that E16 blocks membrane fusion of WNV, we evaluated the fusogenic properties of WNV in a model liposome system. To this end, WNV particles were metabolically labeled with pyrene hexadecanoic acid and purified by density gradient centrifugation. Subsequently, pyrene-labeled virions were pre-incubated with various concentrations of E16, E60 or E111 (a DIII-specific non-neutralizing control MAb [24]) and mixed with liposomes. The mixture was acidified to pH 5.4 and fusion was measured on-line in a fluorimeter at 37°C as a function of the decrease in pyrene excimer fluorescence. WNV fuses rapidly and efficiently with liposomes. In contrast, no membrane fusion activity was measured with saturating concentrations of E16 (Figure 4A). Inhibition of membrane fusion by E16 was dose-dependent as decreasing concentrations of E16 blocked fusion to a lesser degree (Figure 4A and 4B). E111 did not influence the membrane fusion properties of WNV as efficient fusion was measured at all antibody concentrations tested. MAb E60 was observed to induce a dose-dependent inhibition of membrane fusion activity, although a complete inhibition of fusion was not achieved (Figure 4B).

### E16 Fab fragments prevent pH-dependent inactivation of WNV

**Figure 5:**
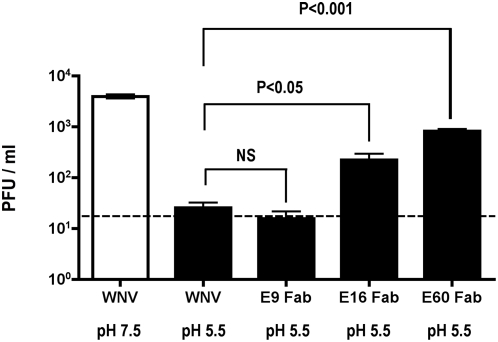
E16 Fab protects WNV from pH-induced inactivation in solution. WNV (3×10^3^ PFU) was incubated alone in the presence of media, 100 μg/ml E16 Fab, E60 Fab or E9 Fab for 30 min on ice. The reaction was diluted 5-fold in media at pH 7.5 or pH 5.5 and incubated at 37°C for 15 min, and then back-neutralized with a 25-fold excess of media at pH 7.5. This mixture was added to a monolayer of Vero cells prior to an overlay with 2% agarose. Three days later, plaques were fixed and scored. The dotted line represents the lower limit of detection the assay (2×10^1^ PFU). Data is expressed as the mean of three separate experiments performed in duplicate. Statistical significance is indicated in the graph and was calculated using a two-tailed paired t test.

Previous studies have shown that exposure of WNV or other flaviviruses to acidic (pH<6) media in the absence of target membranes results in E protein rearrangement, premature exposure of the fusion loop, virus aggregation, and rapid irreversible inactivation of fusion competence [36]–[38]. We reasoned that if E16 neutralized WNV infection by directly blocking the pH-dependent fusion event it should prevent adventitious inactivation in solution after exposure to acidic pH. To test this, WNV (3×10^3^ PFU) was pre-incubated with saturating (100 μg/ml) concentrations of E16, E60, or E9 (a DIII non-neutralizing MAb [24]) Fab fragments. Although the E60 MAb did not appear to enter cells or potently neutralize WNV infection [39], we included this fusion loop-specific Fab as a control because it partially inhibited pH-catalyzed virus fusion in the liposome assay. Excess buffered media at pH 7.5 or pH 5.5 was added to the virus-Fab complexes and incubated at 37°C for 15 min. The solution was normalized after dilution with a 25-fold excess of pH 7.5 media and added to Vero cells for 1 h at 37°C to allow infection as the monovalent Fab fragments detached. As expected, exposure to a pH 7.5 solution did not change WNV infectivity, as the monolayer contained ∼3.9×10^3^ PFU (Figure 5). In contrast, treatment with a pH 5.5 solution inactivated WNV and reduced infectivity (P<0.0001) below the limit of detection (∼20 plaques). The E9 Fab failed to protect the virus from low pH inactivation, whereas neutralizing concentrations of E16 and E60 Fabs at pH 5.5 partially protected WNV from pH-induced inactivation as 2.2 and 8.2×10^2^ PFU were detected, respectively (Figure 5; P<0.05 and P<0.0001).

Because less infectious virus was detected with E16 compared to E60 treatment following pH normalization and dilution, we hypothesized that even a small number of bound E16 Fab could still inhibit infectivity since this MAb requires a low fractional occupancy for efficient neutralization [26]. Conversely, even detachment of a few E60 Fabs could significantly increase infectivity because virtually complete occupancy is required for neutralization by this MAb [40]. Experiments were repeated and excess recombinant E protein DIII (0.4 mg/ml) was added at the time of pH normalization and dilution to compete off additional bound E16 Fab. The addition of excess recombinant DIII further increased WNV infectivity by ∼4 fold (data not shown), presumably by lowering the number of bound E16 Fab on some virions below the threshold for neutralization. Overall, these experiments show that saturating concentrations of both E16 and E60 Fabs at least, partially prevent irreversible pH-dependent inactivation of WNV in the absence of target membranes.

## Discussion

Antibody neutralization is essential for protection against infection by many viruses. A greater understanding of the mechanism(s) by which the most strongly neutralizing antibodies act could facilitate strategies for generating targeted vaccines and immunotherapies. To establish the mechanism of action of E16, a strongly neutralizing anti-WNV MAb with therapeutic potential, we performed a series of cellular and biochemical experiments. Cell biology studies demonstrate that E16 blocks WNV infection at a post-entry stage by sequestering the virus in acidic compartments and preventing its egress into the cytoplasm. Biochemical experiments demonstrate that E16 neutralizes WNV by directly blocking the pH-dependent fusion process. Thus, the inhibitory activity of E16 against WNV in vivo is likely defined by its ability to block viral fusion and nucleocapsid penetration into the cytoplasm where replication occurs.

Analysis of the crystal structure of E16 Fab bound to WNV E protein led to a hypothesis that E16 blocked the structural rearrangement required for fusion at low pH [27]. Indeed, E16 engages a large solvent-exposed surface of DIII, a domain that is positioned distinctly in the pre- and post-fusion E protein conformations [10]. The biochemical data presented here demonstrating that E16 Fab blocks the pH-dependent inactivation of WNV in solution is consistent with a direct inhibition of the structural transition of E protein that occurs during fusion. Nonetheless, definitive evidence of this structural mechanism awaits solution of the E16-WNV structure by cryo-electron microscopy in media at acidic pH.

In surface plasmon resonance (SPR) binding studies, E16 bound DIII of the WNV E protein with similar affinity across a range of pH values from pH 5 to pH 8 (B.S. Thompson, M.S. Diamond and D.H. Fremont, unpublished data). This explains why the binding and neutralizing activity of E16 is not altered as the virus-MAb complex transits through the endosomal compartments. Indeed, the confocal microscopy experiments showed co-localization of E16 and virus through acidic compartments into the lysosome. Our investigations with MAbs are consistent with an earlier study showing a strongly neutralizing polyclonal serum against WNV inhibited at a post-attachment step [41]; the authors of that study speculated but did not show that the most potently inhibitory antibodies block viral fusion. One reason why antibody blockade of fusion may be particularly potent in vivo for flaviviruses is because it acts downstream of an increasing number of cellular attachment factors (e.g., DC-SIGN, DC-SIGNR, heparin sulfate, Fc-γ receptors, and α_v_β_3_ integrin [42]–[45]).

The confocal microscopy experiments also suggest that E16-opsonized WNV is retained in acidic compartments that are ultimately targeted for degradation. Antibodies like E16 that block fusion may be particularly potent at clearing viral infection in vivo because in addition to directly limiting transit to and replication in the cytoplasm they effectively convert permissive cells into ones that target virus for destruction. This feature of E16, along with its ability to disrupt transneuronal spread [46], high affinity, and capacity to neutralize at low virion occupancy [26], begins to explain its single-dose potent post-exposure therapeutic activity in animals [24],[47].

**Figure 6:**
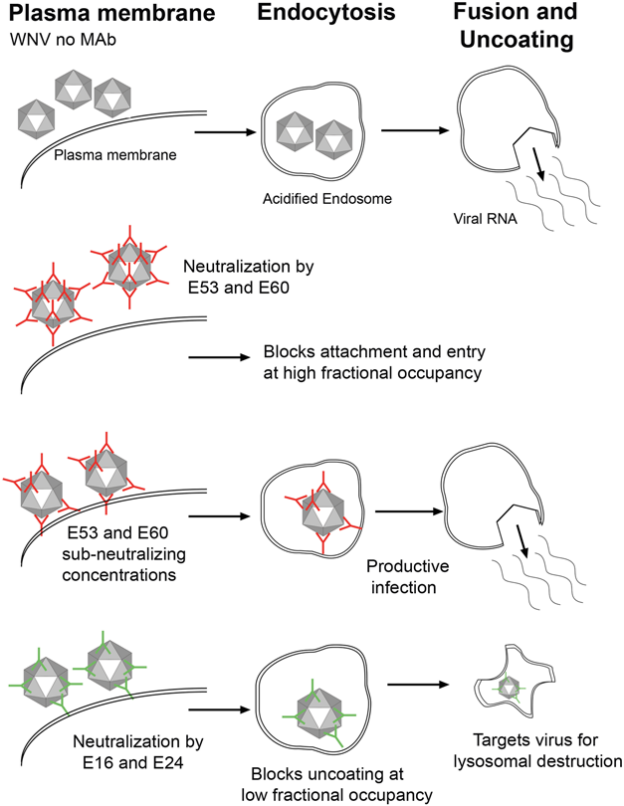
Model of anti–WNV MAb neutralization. The neutralization of WNV infection in Vero cells occurs by different mechanisms depending on the epitopes occupied by the MAbs. WNV infection in the absence of antibodies results in attachment, endocytosis, fusion, uncoating and release of the viral RNA into the cytoplasm. Vero cell infection in the presence of neutralizing concentrations of the fusion loop MAbs E53 or E60 results in a blockade in viral attachment. In contrast, infection in the presence of E53 or E60 at sub-neutralizing concentrations allows for efficient attachment, entry, fusion and infection. Infection in the presence of neutralizing concentrations of E16 or E24 (which require lower fractional occupancy for neutralization) results in relatively normal attachment and endocytosis. However, these MAbs inhibit fusion of the viral membrane with the endosomal membrane leading to subsequent targeting of the virus particles to the lysosome.

The mechanistic analysis of E16 and WNV is supported by recent studies with MAbs against DIII of TBEV, some of which also blocked fusion of pyrene-labeled virus with liposomes [48]. Nonetheless, it remains unclear if the DIII MAbs against TBEV have equivalent neutralizing capacity and bind the same structural epitope as E16. The TBEV study also showed that DII-fusion loop MAbs were effective at blocking liposomal fusion. Although we also observed efficient dose-dependent inhibition of membrane fusion with E60, approximately one-third of the virus particles remained fusion competent even under conditions of antibody excess. This data is consistent with our observation that E53 and E60 are less strongly inhibitory MAbs against WNV [39] and that heterogeneity of WNV particles with respect to their state of maturation (mostly immature, partially mature, or fully mature) affects the ability of fusion loop MAbs to bind and neutralize infection [40]. As the fusion loop epitope is poorly accessible on the mature WNV virion [13],[40],[49], E53 and E60 MAbs require a relatively high fractional occupancy to inhibit infection [40]. Indeed, they may not achieve sufficient MAb concentration in the endosomes to neutralize by this mechanism. Instead, at least for Vero cells, our data with E53 and E60 suggests that antibodies of this class block at a proximal attachment step [27]. Based on these observations, we have developed a model for how the DII-fusion loop and DIII-lateral ridge MAbs neutralize WNV infection (Figure 6).

Blockade of viral fusion by antibodies or pharmacologic agents is usually considered as a therapeutic strategy for viruses that fuse with the plasma membrane. For example, enfuvirtide (Fuzeon™ or T-20 peptide) effectively inhibits entry of HIV at the plasma membrane of CD4^+^ T cells by interfering with the requisite structural transition that brings viral and cell surfaces into proximity for fusion (reviewed in [50]). In contrast, there have been relatively few descriptions of antibodies that neutralize flaviviruses by blocking endosomal fusion. Butrapet et al described an anti-Japanese encephalitis virus antibody (MAb 503) that inhibited fusion-induced syncytia of infected insect cells and virus internalization into Vero cells. Although they concluded that this MAb functioned at a step post-attachment, they did not clearly demonstrate that it directly blocked fusion [51]. Similarly, the mechanism of action of the potently neutralizing anti-DENV2 MAb, 3H5-1 [52], has been speculated. Whereas He et al, showed that 3H5-1 blocked attachment of DENV2 to Vero cells [53], Se-Thoe et al, using LLC-MK2 cells, concluded that 3H5-1 primarily blocked the DENV2 fusion at the plasma membrane [54]. We recently localized the epitope of 3H5-1 of DENV2 to residues in the N-terminal region and FG loops of the lateral ridge of DIII, in an analogous position to that for E16 and WNV DIII [55]. Although further studies are necessary, based on structural localization and functional potency, we speculate that 3H5-1 and other strongly neutralizing DIII lateral ridge MAbs inhibit flavivirus infections, at least in part through similar fusion blocking mechanisms.

In summary, our experiments define the mechanism of action of a potently inhibitory therapeutic antibody against WNV. E16 prevents egress of WNV from endosomes, leading to retention in progressively acidic compartments and likely destruction in the lysosome. Vaccines that skew the immune response towards production of antiviral antibodies that inhibit fusion may improve protection against challenge. For highly promiscuous viruses like flaviviruses, targeting of the endosomal fusion step may be particularly relevant given the discovery of increasing numbers of distinct entry pathways on mammalian cells [42],[43].

## Materials and Methods

### Cell culture and propagation of WNV

Vero cells were used for confocal microscopy experiments, the plasma membrane fusion assay, and to titrate infectious virus by plaque assay. Vero cells were grown in Dulbecco's modified eagle's medium (DMEM) supplemented with 10% FBS, 10 mM HEPES and 1% penicillin/streptomycin, as described [56]. WNV (strain 3000.0259, New York, 2000) [57] was propagated in C6/36 *Aedes albopictus* cells, aliquotted, and frozen at −80°C.

### Pyrene-labeled virus particles

Pyrene-labeled WNV was isolated from the medium of infected BHK21 cells that was cultured in the presence of 15 μg/ml of 16-(1-pyrenyl)-hexadecanoic acid (Invitrogen, Breda, The Netherlands), essentially as described before for alphaviruses [58],[59]. BHK21 cells were infected at a MOI of 4. At 24 h post-infection, the supernatant was harvested and pyrene-labeled WNV particles were pelleted by ultracentrifugation (Beckman type 19 rotor; 15 hr at 48,500×g at 4°C). Subsequently, the virus particles were purified on an Optiprep (Axis-Shield, Oslo, Norway) density (15–55% w/v) gradient by ultracentrifugation (Beckman SW41 rotor; 18 hr at 100,000×g at 4°C). The infectivity of the virus preparation was determined by titration on BHK21-15 cells. Protein concentration was determined by micro-Lowry analysis.

### Preparation of liposomes

Large unilamellar vesicles were prepared by a freeze/thaw extrusion procedure as described [59]. Liposomes consisted of phosphatidylcholine (PC) from egg yolk, phosphatidylethanolamine (PE) prepared by transphosphatidylation of egg PC, and cholesterol in a molar ratio of 1:1:2. Liposomes were prepared with an average size of 200 nm. All lipids were obtained from Avanti Polar Lipids (Alabaster, AL).

### Antibodies and flow cytometry

The anti-WNV antibodies E9, E16, E24, E53, E60, and E111 have been previously described [24],[27],[39]. Fab fragments were generated by papain digestion and purified by protein A affinity and size exclusion chromatography as described [27]. The generation and purification of the E16 scFv will be described in detail elsewhere (B. Kauffman, S. Johnson, D. Fremont, M. Diamond, and M. Rossmann, manuscript in preparation). Direct conjugation of MAbs to fluorochromes was performed using an Alexa Fluor® 488 (or 647) MAb labeling kit (Invitrogen, Carlsbad, CA) according to the manufacturer's instructions. Both anti-human and anti-mouse secondary antibodies conjugated to fluorochromes were purchased (Invitrogen) and used at a 1:200 dilution for confocal microscopy and flow cytometry. Flow cytometric analysis was performed using a BD FACS Calibur and BD Cellquest Pro™ software (Becton Dickinson, San Jose, CA).

### Virus binding assays and confocal microscopic analysis

Vero cells were plated at ∼7,500 cells/well in 8-well Lab-Tek chambered slides (Nunc, Rochester, NY) and incubated overnight. The cells were infected with WNV (MOI of 100) in the presence or absence of Alexa-488 conjugated antibodies at the indicated temperature and time, washed with PBS, and fixed with 2% paraformaldehyde in PBS for 30 min at room temperature. Acidified endosome and lysosome compartments were identified with Lysotracker red (Invitrogen) by adding the dye (50 nM) to the cells for the last 30 min of the incubation prior to fixation.

To assess whether blockade of endosomal acidification mimics treatment with E16, Vero cells were infected at an MOI of 100 in the presence of 10 nM concanamycin A1 or 100 μg/ml hE16 for 3 h or 24 h, fixed with 2% paraformaldehyde, and permeabilized with PBS supplemented with 0.1% saponin. Cells were stained with a pool of Alexa-488 conjugated mouse anti-E MAbs and in some experiments, Alexa-647-conjugated goat anti-human IgG. After extensive washing and fixation, cells were analyzed by confocal microscopy using a Zeiss LSM510 META Laser Scanning Confocal Microscope (Carl Zeiss Inc., Thornwood, NY) as described [60]. Images were analyzed using the LSM510 software suite and Volocity™ software package (Improvision Inc., Waltham, MA).

### Plasma membrane fusion assay

The assay for plasma membrane fusion of flaviviruses has been described previously [32]. We adapted the protocol to test the effects of MAbs on WNV fusion at the plasma membrane. Briefly, Vero cells were plated in 12 well plates at 5×10^4^ cells per well and incubated for 24 h at 37°C. The cells were then pre-incubated with 10 nM concanamycin A1 for 30 min. WNV (MOI of 100) was complexed with 100 μg/ml E16 IgG, E16 scFv, E60 IgG or control medium for 30 min at 4°C and bound to Vero cells for 2 h on ice. Subsequently, cells were washed twice with iced PBS and pre-warmed DMEM (buffered to pH 5.5 or pH 7.5) was added at 37°C for ∼7 min. The cells were then washed with PBS and incubated for 24 h at 37°C in DMEM containing 10 nM concanamycin A1, which blocks virus fusion after receptor mediated entry pathways. The cells were washed twice in PBS and fixed in PBS with 2% paraformaldehyde, permeabilized with 0.1% saponin and stained with an oligoclonal pool of Alexa Fluor-488-labeled anti-WNV MAbs. Samples were processed by flow cytometry and data was analyzed using the Cellquest Pro™ software.

### pH inactivation assay in solution

WNV (∼3×10^3^ PFU) was incubated alone or with 100 μg/ml E16 Fab, E60 Fab or E9 Fab in DMEM at neutral pH for 30 min at 4°C. The reactions were then diluted 5-fold in DMEM supplemented with 20 mM succinic acid (pH 5.5) or 20 mM HEPES (pH 7.5) and incubated at 37°C for 15 min. Each reaction was subsequently neutralized by a 25-fold dilution in DMEM at pH 7.5 and added to a monolayer of Vero cells in a 6 well plate for 1 h at 37°C. Following this incubation, the cells were overlaid with 2% low melting agarose and a standard plaque assay was performed. In some experiments, recombinant DIII (0.4 mg/ml) purified from *E. coli* [27] was added at the time of 25-fold dilution to compete bound Fabs.

### WNV–liposome fusion assay

Fusion of pyrene-labeled WNV with PE/PC/cholesterol (molar ratio of 1:1:2) liposomes was monitored continuously in a Fluorolog 3–22 fluorometer (BFi Optilas, Alphen aan den Rijn, The Netherlands), at excitation and emission wavelengths of 345 nm and 480 nm. Pyrene-labeled WNV (0.35 μg protein; corresponds to 1.5×10^10^ particles) and an excess of liposomes (140 nmol phospholipid; corresponds to 3×10^10^ liposomes) was mixed in a final volume of 0.665 ml in 5 mM HEPES pH 7.4, 150 mM NaCl, and 0.1 mM EDTA. The content was stirred magnetically at 37°C. At t = 0 sec, the pH of the medium was adjusted to 5.4 by addition of 35 μl 0.1 MES, 0.2 M acetic acid, pre-titrated with NaOH to achieve the final desired pH. The fusion scale was calibrated such that 0% fusion corresponded to the initial excimer fluorescence value. The 100% value was obtained through the addition of 35 μl 0.2 M octaethyleneglycol monododecyl ether (Fluka Chemie AG, Buchs, Switzerland) to achieve an infinite dilution of the probe. The extent of fusion was determined 60 seconds after acidification. To analyze the influence of E16, E60, and E111 on WNV fusion, pyrene-labeled WNV was incubated with increasing concentrations of MAbs for 1 hr at 20°C prior to mixing with liposomes.

## Supporting Information

Figure S1 Cells were infected with WNV in the absence of MAbs for (A) 3, (B) 6, and (C) 24 hours as indicated, fixed, stained with an oligoclonal mixture of anti-E MAbs, and analyzed by confocal microscopy. Representative images are shown from one of at least four independent experiments.
(3.22 MB TIF)
