# Supplementary figures and images for: A Therapeutic Antibody against West Nile Virus Neutralizes Infection by Blocking Fusion within Endosomes
Source: PLoS Pathog. 2009 May 29;5(5):e1000453. doi: 10.1371/journal.ppat.1000453 (PMC2679195; doi:10.1371/journal.ppat.1000453)

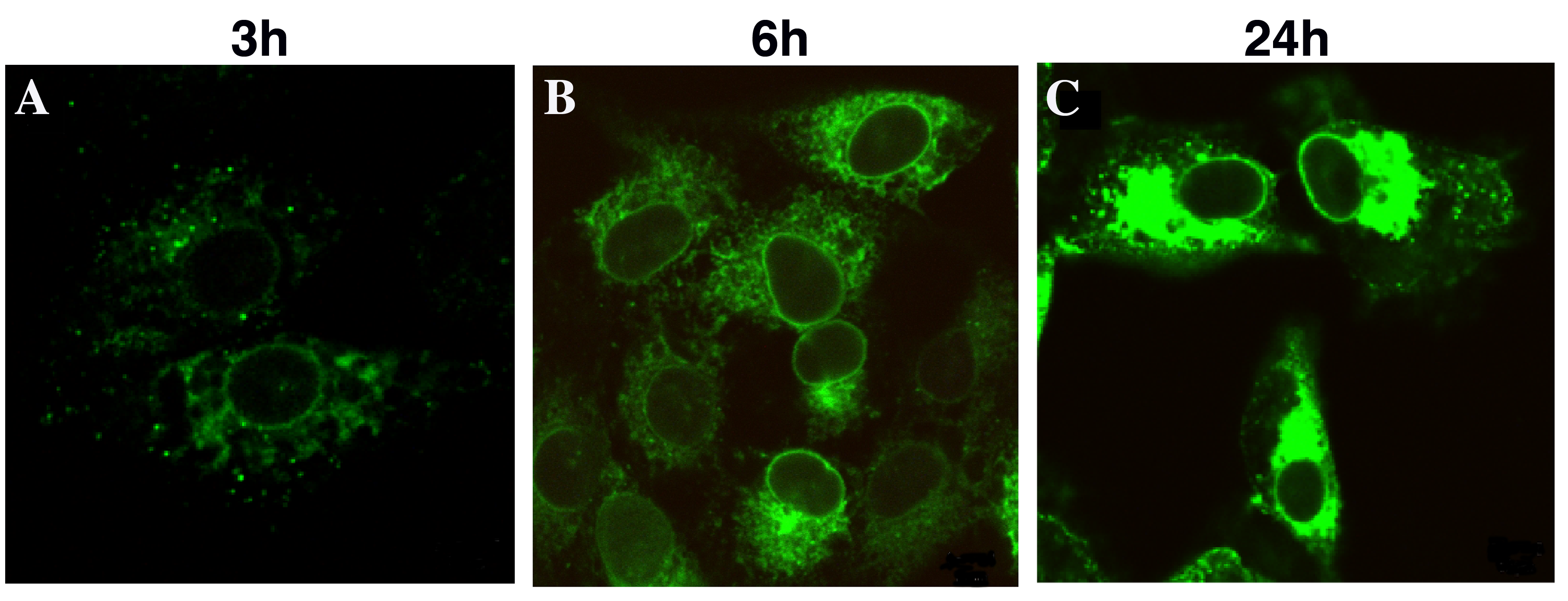

Supplement: Figure S1 — Cells were infected with WNV in the absence of MAbs for (A) 3, (B) 6, and (C) 24 hours as indicated, fixed, stained with an oligoclonal mixture of anti-E MAbs, and analyzed by confocal microscopy. Representative images are shown from one of at least four independent experiments. (3.22 MB TIF) [file ppat.1000453.s001.tif]
